# Ultrasound assessment and scoring of tendinopathy in hemophilia: Development of the Tendon Haemophilic Damage ‐ Ultrasound (THD‐US) method

**DOI:** 10.1002/jcu.23845

**Published:** 2024-09-20

**Authors:** Danilo Donati, Flavio Origlio, Stefano Galletti, Marco Miceli, Paolo Spinnato, Lelia Valdrè, Lydia Piscitelli, Vincenzo Ricci, Giuseppina Mariagrazia Farella, Fabio Vita, Roberto Tedeschi, Maria Grazia Benedetti

**Affiliations:** ^1^ Physical Therapy and Rehabilitation Unit Policlinico di Modena Modena Italy; ^2^ Clinical and Experimental Medicine PhD Program University of Modena and Reggio Emilia Modena Italy; ^3^ Physical Therapy and Rehabilitation Unit IRCCS‐Rizzoli Orthopedic Institute, University of Bologna Bologna Italy; ^4^ Musculoskeletal Ultrasound School Italian Society for Ultrasound in Medicine and Biology Bologna Italy; ^5^ Department of Diagnostic and Interventional Radiology RCCS Istituto Ortopedico Rizzoli Bologna Italy; ^6^ Haematology Department Inherited Bleeding Disorders Unit, IRCCS AOUBO Bologna Italy; ^7^ Physical and Rehabilitation Medicine Unit Luigi Sacco University Hospital, ASST Fatebenefratelli‐Sacco Milan Italy; ^8^ IRCCS Istituto Ortopedico Rizzoli 1st Orthopaedics and Traumatology Clinic Bologna Italy; ^9^ Department of Biomedical and Neuromotor Sciences, Alma Mater Studiorum University of Bologna Bologna Italy

**Keywords:** hemophilia, scoring system, tendinopathy, THD‐US, ultrasonography

## Abstract

This study aimed to develop and validate the tendinopathy hemophilia detection with ultrasonography (THD‐US) protocol for assessing hemophilia‐related tendinopathy. Twenty male patients with hemophilic arthropathy underwent ultrasound evaluations of 200 tendons. The THD‐US scoring method assessed structural changes, hyperemia, and calcifications, revealing various tendon abnormalities. This protocol provides a standardized, efficient method for assessing tendinopathy in hemophilia patients, potentially improving patient management and outcomes.

## Graphical abstract

This study introduces the THD‐US protocol, a novel ultrasound‐based scoring system for assessing tendinopathy in hemophilia patients. It effectively identifies and grades tendon abnormalities, facilitating better management and treatment strategies in clinical practice.

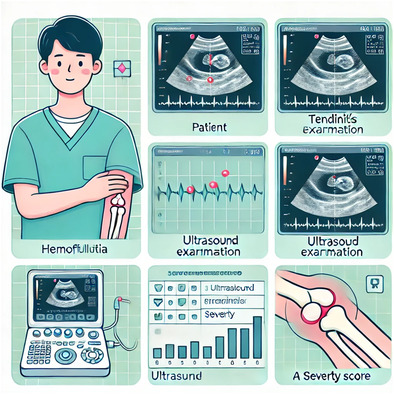

## 1 BACKGROUND

Hemophilia, characterized by recurrent bleeding episodes, manifests primarily as hemophilia A (factor VIII deficiency) and hemophilia B (factor IX deficiency), both transmitted through the X chromosome. Intra‐articular bleeding (hemarthrosis) commonly affects major joints like the elbow, knee, and ankle, leading to complications such as degenerative arthropathy.^1^, ^2^ Haemophilic arthropathy is a result of the development of recurrent hemarthrosis in the same target joint.^3^ Synovitis in HA, associated with iron deposition, leads to synovial inflammation and joint damage. Recurrent hemarthrosis triggers inflammatory responses within the joint, leading to acute and then chronic synovitis, which subsequently results in haemophilic arthropathy characterized by degenerative and destructive joint changes. Periarticular structures, including tendons and surrounding soft tissues, are often involved due to the chronic inflammatory environment created by recurrent hemarthrosis, leading to their degeneration and contributing to musculoskeletal limitations.^1^ Diagnostic imaging, particularly ultrasound, is crucial for assessing joint and tendon damage in hemophilia patients. This joint damage often extends to surrounding tendons, leading to tendinopathy, which is commonly observed in hemophilia patients.^4^ It is important to note that hemophilia‐induced tendinopathy significantly differs from mechanical tendinopathy caused by overuse, as it results from recurrent bleeding and inflammation, affecting tendon structure differently.^5^ This study aimed to develop the tendinopathy hemophilia detection with ultrasonography (THD‐US) protocol for assessing hemophilia‐related tendinopathy and to provide preliminary insights into its application.^2^, ^6^

## 2 METHODS

**FIGURE 1:**
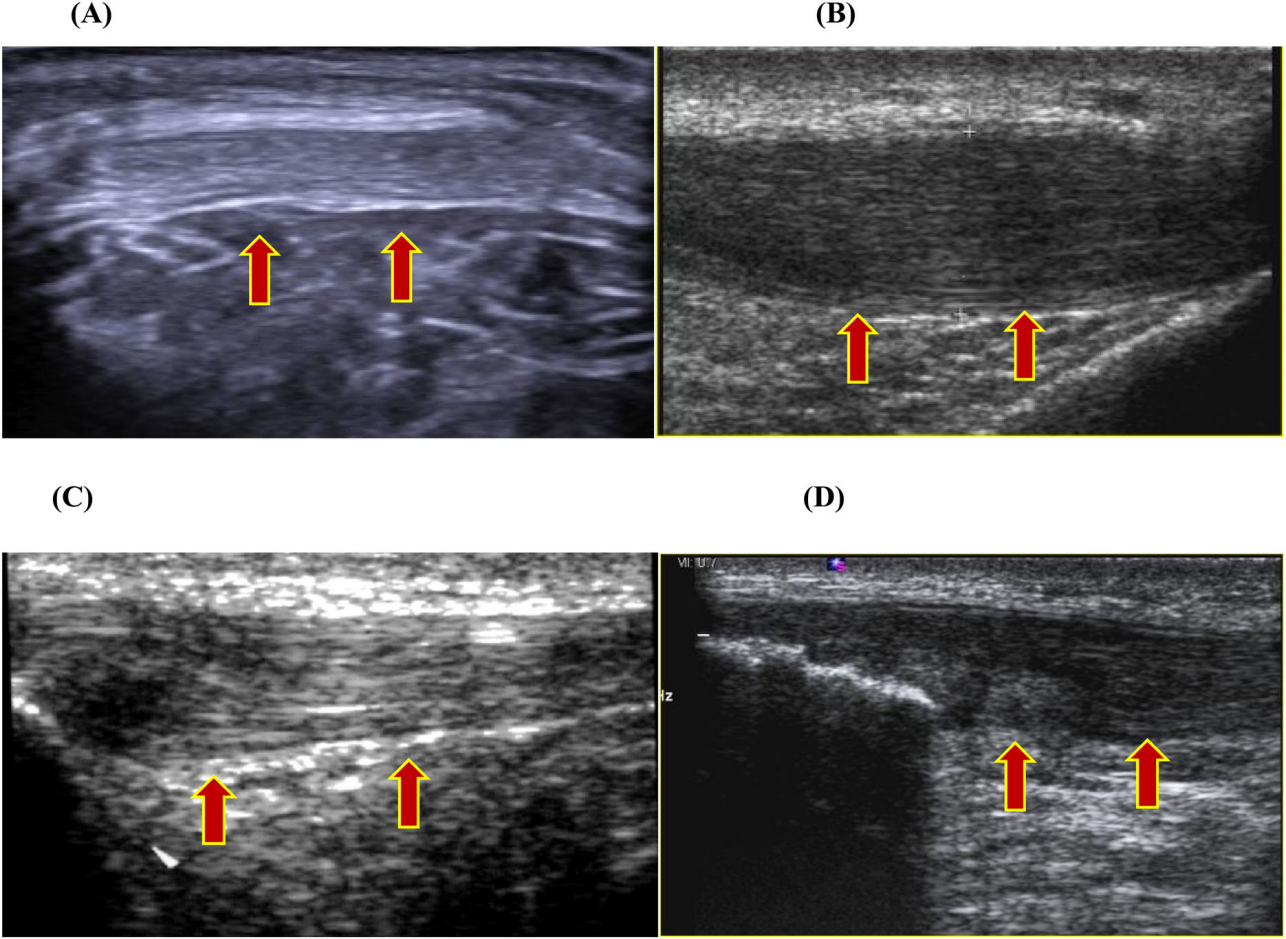
Echogenicity and eco‐structural alteration: (A) grade 0 normal tendon, (B) grade 1—mild ecostructure alteration, (C) grade 2—moderate ecostructure alteration, and (D) grade 3—severe ecostructure alteration and degeneration.

**FIGURE 2:**
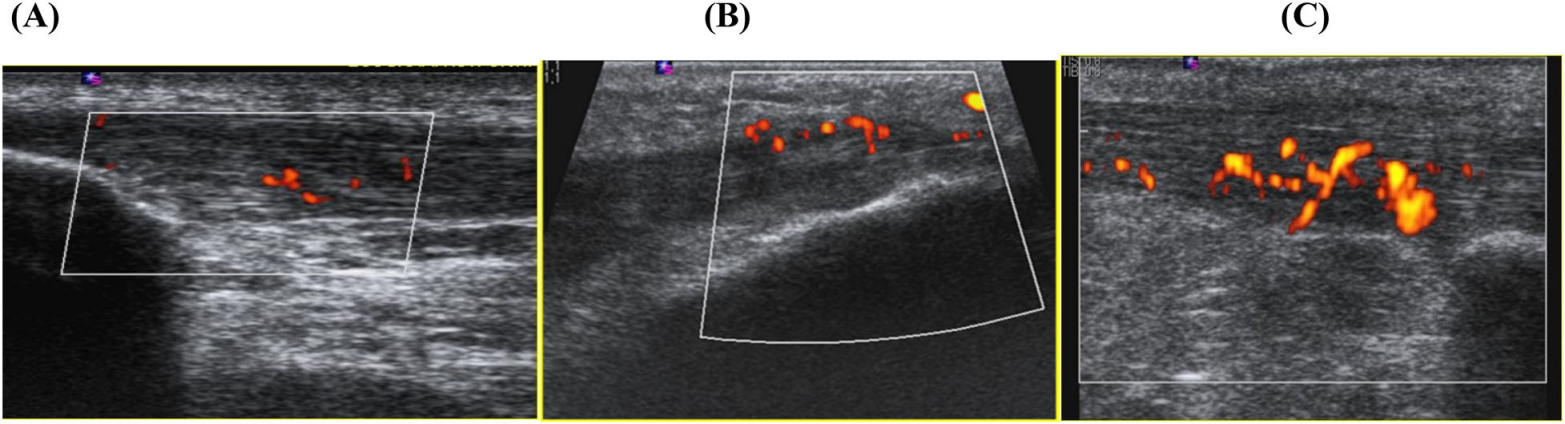
Signs of hyperemia on power‐color Doppler: (A) grade 1–1 to 2 new vessels, (B) grade 2—few vessels/low blood flow, and (C) grade 3—many vessels/considerable blood flow.

**FIGURE 3:**
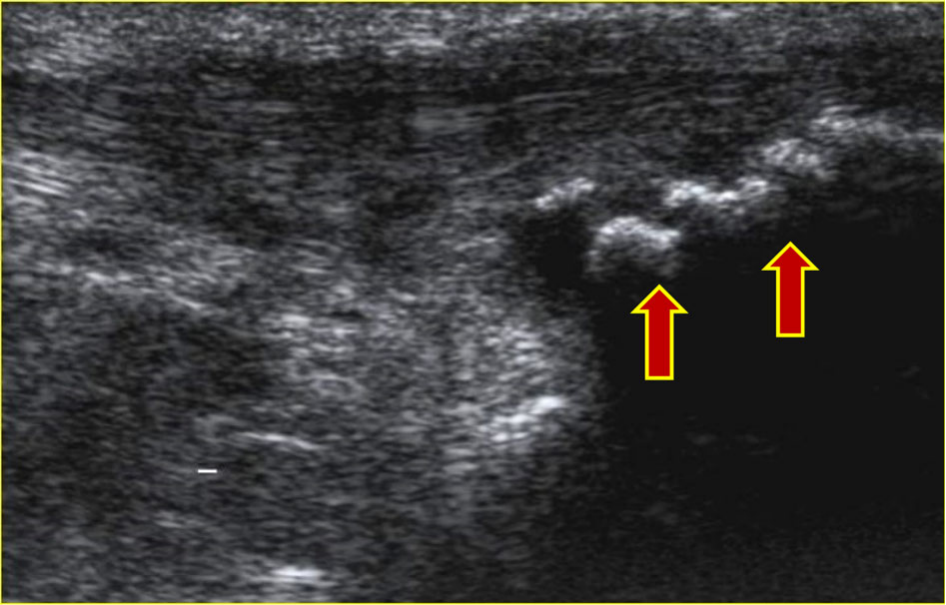
Presence of the insertional calcifications on the tendon.

**FIGURE 4:**
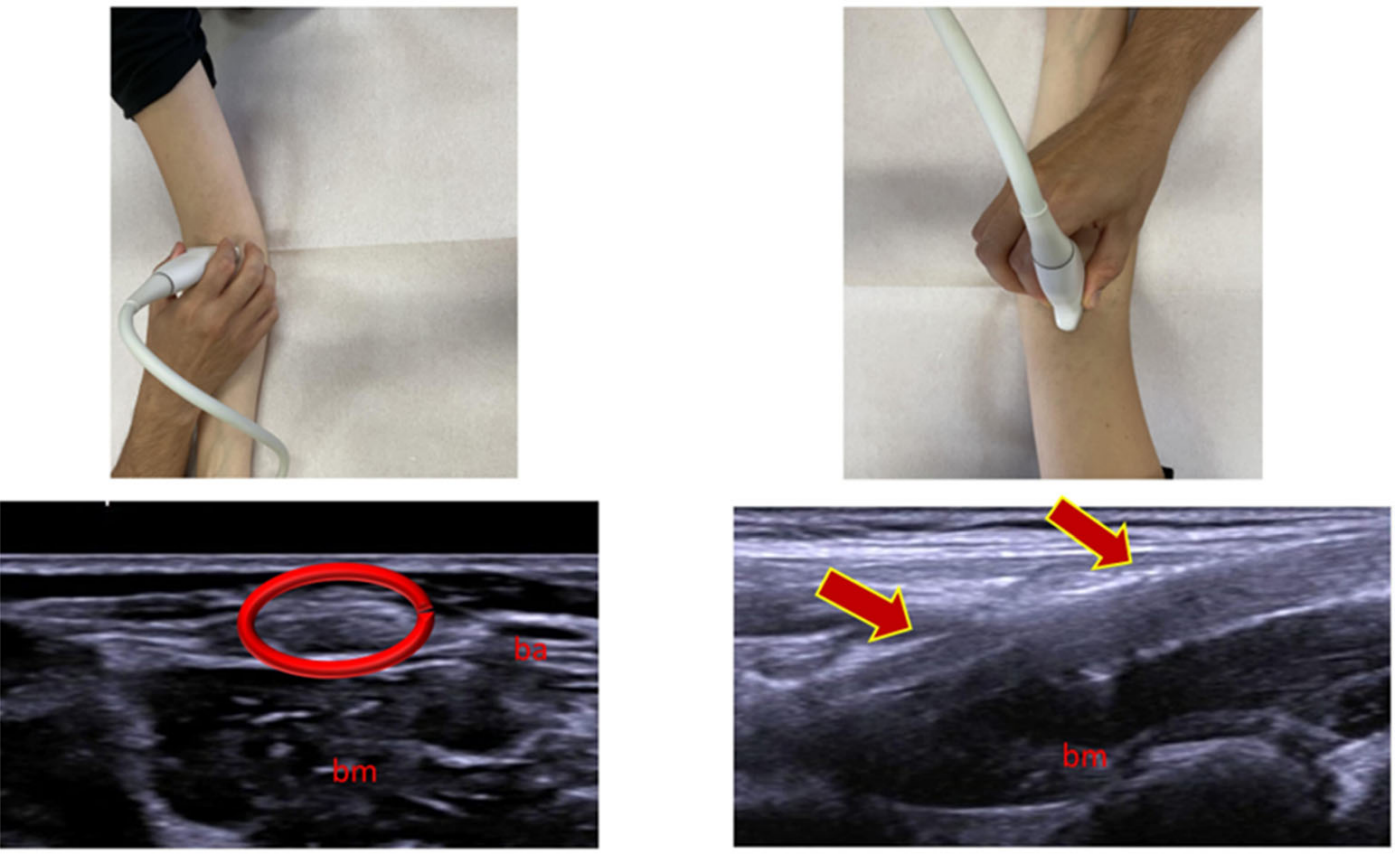
Ultrasound image acquisition points and corresponding anatomical structures. Top row: Photographs showing the positioning of the ultrasound probe for scanning the distal biceps brachii tendon (left) and the distal triceps brachii tendon (right). Bottom row: Corresponding ultrasound images of the tendons. The left image (circled in red) shows the biceps brachii tendon (ba) and brachialis muscle (bm). The right image (indicated by arrows) shows the triceps brachii tendon (bm: Brachialis muscle).

Participants with hemophilic arthropathy from the Center for Congenital Hemorrhagic Diseases. The study included male participants aged 18–65 years with ultrasound signs of hemophilic arthropathy. Exclusion criteria were elbow, knee, and ankle arthroplasty, or synovectomy. Ultrasound assessments targeted the biceps and triceps brachii tendons at the elbow, quadriceps and patellar tendons at the knee, and Achilles tendons at the ankle. A consensus between an experienced musculoskeletal radiologist and a specialist in physical medicine and rehabilitation (physiatrist) was maintained to ensure unbiased evaluation. The THD‐US protocol scoring method assessed structural changes in echogenicity, hyperemia on power‐color Doppler, and the presence of calcifications, with scores ranging from 0 to 7 (Table 1). The ultrasound examination was performed with a linear probe with musculoskeletal presets for elbow's tendons evaluation (7–16 MHz)^7^, ^8^, ^9^ (Figures 1, 2, 3, 4). The following scanning procedures were performed to assess the elbow, knee, and ankle:

**Elbow**: For the distal biceps brachii tendon (DBBT), the elbow was fully extended and the hand supinated. The DBBT was observed laterally to the brachial artery and superficial to the brachialis muscle. For the distal triceps brachii tendon, the elbow was flexed at 90°, and the tendon was assessed using a sagittal scan.
**Knee**: The quadriceps tendon was evaluated along a sagittal plane proximal to the patella with slight knee flexion to reduce anisotropy. The patellar tendon was observed inferiorly below the patella within a sagittal plane.
**Ankle**: The patient assumed a prone position with the foot positioned outside the examination bed. The Achilles tendon was assessed using a posterior approach with the probe in a sagittal plane.

**TABLE 1:** Tendinopathy haemophilic detection (THD‐US score).

Echogenicity and eco‐structural alteration	Score
Grade 0	0
Grade 1	1
Grade 2	2
Grade 3	3
Signs of hyperemia on power‐color Doppler	
Grade 0—no new vessels visible	0
Grade 1—1–2 new vessels	1
Grade 2—few vessels/low blood flow	2
Grade 3—many vessels/significant blood flow	3
Tendon calcifications	
Absence of calcifications	0
Presence of calcifications	1

*Note*: Degrees of tendinopathy (mild = 1–2, moderate = 3–5, severe = 6–7).

## 3 RESULTS

### 3.1 Participants demographics and tendon evaluations

The study included 20 male participants with hemophilic arthropathy, consisting of varying severities of hemophilia A and B. The mean age of the participants was 40.6 years (SD ± 10.5). Each patient had 10 tendons assessed, resulting in a total evaluation of 200 tendons. Participants were included based on the presence of ultrasound‐detected joint damage, with a minimum score of 2 on the HEAD‐US score to ensure consistent assessment across varying age groups.

### 3.2 Biceps tendons

Upon ultrasound evaluation, the biceps tendons displayed no considerable echostructural alterations, hyperemia, or neovascularization on color Doppler imaging. Additionally, no intratendinous calcifications were detected in the biceps tendons, indicating that these tendons were relatively unaffected by hemophilic arthropathy in this cohort.

### 3.3 Triceps tendons

Notable alterations were observed in the triceps brachii tendons. Specifically, one patient exhibited grade‐three echostructural changes in the left triceps tendon, characterized by severe degeneration and eco‐structural alterations. Another patient showed grade‐three neovascularization in the right triceps tendon, indicating considerable blood flow changes associated with tendinopathy. Furthermore, one subject presented intratendinous calcifications in both triceps tendons, suggestive of advanced tendinopathic changes.

### 3.4 Quadriceps tendons

The quadriceps tendons demonstrated mild to moderate alterations. Grade‐one echostructural changes were observed bilaterally in one subject, indicating early stages of tendinopathy. However, there were no considerable changes in color Doppler signal, implying minimal vascular involvement. Bilateral insertional tendon calcifications were also noted in one patient, reflecting localized degenerative changes at the tendon‐bone interface.

### 3.5 Patellar tendons

In the patellar tendons, no considerable echostructural alterations were identified. However, one subject exhibited grade‐one neovascularization with color Doppler in the left patellar tendon, indicating mild vascular changes. Intratendon calcifications were present bilaterally in one subject, highlighting degenerative changes within the tendons.

### 3.6 Achilles tendons

The Achilles tendons presented with varying degrees of abnormalities. One subject showed grade‐one echostructural alterations in the right Achilles tendon, indicating early tendinopathic changes. Another subject exhibited grade‐one positive color Doppler signals in the right Achilles tendon, reflecting mild hyperemia. Bilateral insertional tendon calcifications were observed in one subject, suggesting localized degenerative processes at the Achilles tendon insertions.

### 3.7 THD‐US scores

The distribution of THD‐US scores across the evaluated tendons is summarized in Table 2. This table highlights the frequency of each score combination for echogenicity, hyperemia, and calcifications, providing a detailed overview of the tendinopathic changes observed in hemophilia patients.

**TABLE 2:** Frequency distribution of THD‐US scores across different tendon locations in hemophilia patients.

Tendon location	Echogenicity (0–3)	Hyperemia (0–3)	Calcifications (0–1)	Total THD‐US Score	Frequency
Biceps tendons	0	0	0	0	20
Triceps tendons	3	3	1	7	1
Triceps tendons	3	1	1	5	1
Quadriceps tendons	1	0	1	2	1
Patellar tendons	0	1	1	2	1
Achilles tendons	1	1	1	3	1

The table presents the frequency distribution of THD‐US scores across various tendon locations (Biceps, Triceps, Quadriceps, Patellar, and Achilles) in hemophilia patients. The scores for echogenicity (0–3), hyperemia (0–3), and calcifications (0–1) are summed to obtain the total THD‐US score. The frequency column indicates the number of tendons with each specific scoring combination observed in the study.

### 3.8 Summary of findings

- **Biceps tendons**: No considerable echostructural alterations, hyperemia, neovascularization, or calcifications.
- **Triceps tendons**: Considerable findings included severe echostructural changes, neovascularization, and calcifications in specific cases.
- **Quadriceps tendons**: Mild echostructural changes and calcifications noted.
- **Patellar tendons**: Minimal neovascularization and bilateral calcifications observed.
- **Achilles tendons**: Mild echostructural alterations, hyperemia, and calcifications detected.

### 3.9 Implications

These findings underscore the variability in tendon involvement in hemophilic arthropathy, with certain tendons like the triceps and Achilles showing more pronounced changes compared to others such as the biceps. The THD‐US protocol demonstrated its utility in identifying and scoring tendinopathy severity, providing valuable insights for targeted patient management and treatment strategies.

The detailed results highlight the effectiveness of the THD‐US method in capturing subtle and considerable tendinopathic changes, thereby reinforcing its potential application in routine clinical practice for hemophilia participants.

## 4 DISCUSSION

This study highlights the potential of the THD‐US scoring system to standardize tendinopathy assessment in hemophilia participants. The THD‐US scanning protocols were designed to be accessible to non‐expert musculoskeletal sonographers, compatible with standard US machines, informative for joint status, reliable for monitoring treatment efficacy, and time‐efficient for clinical implementation. Future studies should explore the accuracy and effectiveness of the THD‐US score in longitudinal evaluations and its integration with other imaging modalities for comprehensive tendon assessment. The study provides considerable insights into tendon abnormalities in hemophilia participants. The THD‐US scoring method, which evaluates structural changes in echogenicity, hyperemia, and calcifications, offers a comprehensive assessment of tendinopathy. The absence of considerable changes in biceps tendons suggests varying susceptibility of different tendons to hemophilic arthropathy. The notable alterations in triceps, quadriceps, patellar, and Achilles tendons emphasize the importance of targeted ultrasound evaluations in these participants. This method aims to simplify examination and interpretation while ensuring strong intra‐ and inter‐reader reliability. The integration of THD‐US into routine hemophilia care could facilitate efficient evaluation and scoring of tendinopathy activity and damage, ultimately improving patient management and outcomes. Additionally, a comparison between ultrasound findings and clinically reported severity using validated outcomes, such as the TENDINS‐A, would provide further insights into the accuracy of the THD‐US protocol.^10^ Future studies should aim to validate these ultrasound findings against MRI to confirm their consistency.^11^

## FUNDING INFORMATION

The authors received no fundings.

## CONFLICT OF INTEREST STATEMENT

The authors declare no conflicts of interest.

## ETHICS STATEMENT

Ethics Committee (PG n°. 0010368 del 17/06/2021), and all participants included signed the informed‐consent form to participate.

## Data Availability

The data that support the findings of this study are available on request from the corresponding author. The data are not publicly available due to privacy or ethical restrictions.

## References

[1] Roosendaal G , Lafeber FPJG . Blood‐induced joint damage in hemophilia. Semin Thromb Hemost. 2003;29:37‐42. 10.1055/s-2003-37938 12640563

[2] Dunn AL . Pathophysiology, diagnosis and prevention of arthropathy in patients with haemophilia. Haemophilia. 2011;17:571‐578. 21342365 10.1111/j.1365-2516.2010.02472.x

[3] Tedeschi R . Acquired haemophilia a in an elderly patient: a case report of functional recovery through physiotherapy. Int J Surg Case Rep. 2023;110:108769. 37666165 10.1016/j.ijscr.2023.108769 PMC10510055

[4] Poonnoose PM , Hilliard P , Doria AS , et al. Correlating clinical and radiological assessment of joints in haemophilia: results of a cross sectional study. Haemophilia. 2016;22:925‐933. 27385495 10.1111/hae.13023

[5] Scott A , Squier K , Alfredson H , et al. ICON 2019: International Scientific Tendinopathy Symposium Consensus: Clinical Terminology. Br J Sports Med. 2020;54:260‐262. 31399426 10.1136/bjsports-2019-100885

[6] Martinoli C , Della Casa Alberighi O , Di Minno G , et al. Development and definition of a simplified scanning procedure and scoring method for haemophilia early arthropathy detection with ultrasound (HEAD‐US). Thromb Haemost. 2013;109:1170‐1179. 23571706 10.1160/TH12-11-0874

[7] Donati D , Spinnato P , Valdrè L , et al. Ultrasound evaluation of tendinopathy in hemophiliac patients for the purpose of rehabilitation indications. J Clin Med. 2023;12:4513. 37445548 10.3390/jcm12134513 PMC10342756

[8] Matthews W , Ellis R , Furness J , Hing WA . Classification of tendon matrix change using ultrasound imaging: a systematic review and meta‐analysis. Ultrasound Med Biol. 2018;44:2059‐2080. 30007477 10.1016/j.ultrasmedbio.2018.05.022

[9] Cruz‐Montecinos C , Pérez‐Alenda S , Contreras‐Sepúlveda F , Querol F , Cerda M , Maas H . Assessment of tensile mechanical properties of the Achilles tendon in adult patients with haemophilic arthropathy. Reproducibility study. Haemophilia. 2019;25:e27‐e29. 30375147 10.1111/hae.13622

[10] Murphy MC , McCleary F , Hince D , et al. TENDINopathy severity assessment‐Achilles (TENDINS‐A): evaluation of reliability and validity in accordance with COSMIN recommendations. Br J Sports Med. 2024;58:665‐673. 38575200 10.1136/bjsports-2023-107741

[11] Shalabi A , Movin T , Kristoffersen‐Wiberg M , Aspelin P , Svensson L . Reliability in the assessment of tendon volume and intratendinous signal of the Achilles tendon on MRI: a methodological description. Knee Surg Sports Traumatol Arthrosc. 2005;13:492‐498. 16170584 10.1007/s00167-004-0546-0

